# Archaeal Ammonia Oxidizers Dominate in Numbers, but Bacteria Drive Gross Nitrification in *N*-amended Grassland Soil

**DOI:** 10.3389/fmicb.2015.01350

**Published:** 2015-11-30

**Authors:** Anna E. Sterngren, Sara Hallin, Per Bengtson

**Affiliations:** ^1^Microbial Ecology, Department of Biology, Lund UniversityLund, Sweden; ^2^Department of Microbiology, Uppsala BioCenter, Swedish University of Agricultural SciencesUppsala, Sweden

**Keywords:** AOA, AOB, *amoA*, qPCR, ^15^*N*-labeling, nitrogen

## Abstract

Both ammonia-oxidizing archaea (AOA) and ammonia-oxidizing bacteria (AOB) play an important role in nitrification in terrestrial environments. Most often AOA outnumber AOB, but the relative contribution of AOA and AOB to nitrification rates remains unclear. The aim of this experiment was to test the hypotheses that high nitrogen availability would favor AOB and result in high gross nitrification rates, while high carbon availability would result in low nitrogen concentrations that favor the activity of AOA. The hypotheses were tested in a microcosm experiment where sugars, ammonium, or amino acids were added regularly to a grassland soil for a period of 33 days. The abundance of *amoA* genes from AOB increased markedly in treatments that received nitrogen, suggesting that AOB were the main ammonia oxidizers here. However, AOB could not account for the entire ammonia oxidation activity observed in treatments where the soil was deficient in available nitrogen. The findings suggest that AOA are important drivers of nitrification under nitrogen-poor conditions, but that input of easily available nitrogen results in increased abundance, activity, and relative importance of AOB for gross nitrification in grassland soil.

## Introduction

Archaea commonly constitute between 0 and 10% (Timonen and Bomberg, 2009; Bates et al., 2010) of the total prokaryotic abundance in soil, and ammonia-oxidizing archaea (AOA) is the most abundant group of soil archaea (Auguet et al., 2010; Bates et al., 2010). They are known to outnumber the ammonia-oxidizing bacteria (AOB) in multiple environments, particularly in soil, and are suggested to play an important role in soil nitrification (e.g., Leininger et al., 2006; He et al., 2007; Stopnišek et al., 2010). However, factors regulating the relative abundance and importance of AOA and AOB in soil remain unclear. There are, so far, only a few successfully cultivated species of AOA and they possess a variety of characteristics regarding environmental requirements, especially their tolerance to ammonium levels (Könneke et al., 2005; Hatzenpichler et al., 2008; Lehtovirta-Morley et al., 2011; Tourna et al., 2011). For example, some AOA are inhibited by ammonium when it reaches concentrations as low as 2 mM (Hatzenpichler, 2012), while *Nitrososphaera viennensis*, an ammonia-oxidizing archaeon isolated from soil, is inhibited at a ten times higher ammonium concentrations in the growth medium (Tourna et al., 2011). This is low compared to the tolerance levels of 50–1000 mM reported for a range of AOB species (Koops and Pommerening-Röser, 2006). In addition, the affinity for ammonia is higher (more than 200-fold) for the marine archaeon *Nitrosopumilus maritimus* than cultivated AOB (Martens-Habbena et al., 2009), which could suggest niche separation and reduced competition between the AOA and AOB. These findings are reinforced by observations that AOA dominate over AOB in environments where ammonium concentrations are particularly low e.g., seawater or hot springs (Wuchter et al., 2006; Hatzenpichler et al., 2008). Studies from soil also demonstrate that high inputs of ammonium result in an increase in bacterial rather than archaeal *amoA* genes (Di et al., 2009, 2010; Verhamme et al., 2011). This gene encodes the alpha subunit of the enzyme ammonia monooxygenase that catalyze the first step in ammonia oxidation and is often used as a molecular marker to quantify the gene abundance as a proxy for the abundance of the two groups of ammonia oxidizers (Treusch et al., 2005; Leininger et al., 2006; Junier et al., 2010).

A positive relationship between AOA abundance and soil nitrification potential (Yao et al., 2011) or nitrate production (Offre et al., 2009; Gubry-Rangin et al., 2010) has frequently been reported. Moreover, Zhang et al. (2012) found a positive correlation between AOA, but not AOB abundance, and soil nitrate concentration. By contrast, Shen et al. (2008) and Jia and Conrad (2009) showed that AOA dominated numerically over AOB, but that AOB were responsible for ammonia oxidation in an agricultural soil. It has been suggested that AOA may dominate functionally in soil when ammonia is produced at continuous low rates, rather than through addition of high amounts of inorganic fertilizers (Offre et al., 2009; Gubry-Rangin et al., 2010; Levičnik-Höfferle et al., 2012). These sometimes contradictory results might reflect inherent differences among soils in, e.g., organic carbon content, soil particle properties, fertilization regime, and pH (Guo et al., 2013). For example, low pH seems to favor AOA over AOB (He et al., 2012), while the abundance of AOB is positively correlated to soil organic carbon and total nitrogen (Wessén et al., 2011). Another reason to the contradictory findings could be that different approaches have been used to determine nitrification activity. Most studies that have attempted to link the abundance of AOA and AOB to soil nitrification rates have measured net nitrification rates or nitrification potential, and only a few have examined the relationship between gross nitrification rates and AOA abundance (Isobe et al., 2012; Wieder et al., 2013; Prommer et al., 2014). Since nitrogen is added in excess when measuring nitrification potential, the high concentration of substrate is likely to influence the AOA and AOB communities. Furthermore, net nitrification rates poorly predict the gross nitrification rates in soil where nitrate is quickly assimilated (Davidson et al., 1992; Stark and Hart, 1997). Consequently, net nitrification assays do not provide reliable estimates for nitrification activity in soils, and there are even instances where negative correlations between net and gross nitrification rates have been observed (Alves et al., 2013). On the contrary, measurements of gross nitrification rates by means of the ^15^*N*-pool dilution/enrichment technique results in an accurate estimate of microbial nitrification that does not depend on the extent of nitrate consumption occurring during the incubation. The technique is, thus, better suited for linking the abundance of ammonia oxidizers to nitrification rates.

The aim of the present work was to address some of the contradictory findings reported in the literature by determining how gross nitrification rates and the abundance of AOA and AOB responds to recurring additions of sugar, amino acids and ammonium. We hypothesized that input of sugars would result in an increased nitrogen demand of heterotrophic soil microorganisms, creating a nitrogen-poor environment with a low continuous supply of ammonium, which would favor AOA with a high affinity for ammonia, rather than AOB. The opposite scenario was expected as a response to additions of available nitrogen, since there are several reports showing that high concentrations of ammonium favor growth of nitrogen responsive AOB (Di et al., 2009, 2010; Jia and Conrad, 2009). Because these findings can be explained both by a direct inhibitory effect of ammonium on AOA and by poor competitive capabilities of AOA at high substrate concentrations, we also included a treatment where available nitrogen was added as amino acids rather than ammonium. Our hypotheses were tested in a microcosm experiment where gross nitrification rates and abundances of AOA and AOB were determined regularly over a period of 33 days.

## Materials and Methods

### Soil Characteristics

Soil samples were collected from a sandy grassland soil in Vomb, Province of Skåne, Sweden and had a pH value of 6.5 (in a 1:2 mixture of soil:water) and an organic matter content of 4.35% (measured by loss on ignition). The water-holding capacity was 0.3 g water g saturated soil^-1^. The soil was stored at +4°C for 1 month before the experiment started.

### Microcosms and Nutrient Additions

In total, 16 microcosms were set up and each microcosm consisted of 250 g soil stored in a 500-ml transparent, cylindrical polypropylene container with a lid. The microcosms were incubated at room temperature (approximately 20°C) in the dark at 60% water-holding capacity. After an initial stabilization phase of 9 days, the microcosms were subject to four treatments with four replicates each: (1) sugar mix [25 μg carbon (g soil)^-1^], (2) amino-acid mix [25 μg carbon (g soil)^-1^ and 9.6 μg nitrogen (g soil)^-1^], (3) ammonium chloride [9.6 μg nitrogen (g soil)^-1^], and (4) sterile water (control). These amounts were added every 2–3 days for 4.5 weeks (in total 15 additions). The sugar mix consisted of glucose (70% of molar concentration), sucrose (20% of molar concentration) and fructose (10% of molar concentration). The amino-acid mix consisted of 12 amino acids in equal molar concentration (glycine, glutamic acid, glutamine, alanin, asparagin, arginin, histidine, leucine, tryptophan, valine, proline, and serine).

During incubation, the containers were kept closed, but approximately 24 h before addition of the nutrient solutions, the lids were removed to let water evaporate. The evaporated water was replaced to keep the soil at 60% water-holding capacity. Nutrients were added on day 0, 2, 5, 7, 9, 12, 14, 16, 19, 21, 23, 26, 28, 30, and 33. After each addition the soil was thoroughly mixed to ensure a homogenous distribution of the nutrients. Approximately 50 g of soil was sampled from each microcosm, 5, 19, and 33 days after the first addition of nutrients, immediately after nutrient addition and soil mixing. The generation time of the archaeal ammonia oxidizer *N. viennensis* varies between 45 h and 23 days in pure culture, depending on the growth conditions (Tourna et al., 2011). The length of the experiment (33 days) and times of sampling (5, 19, and 33 days after the start of the experiment) should, therefore, be appropriate to capture any changes in the abundance of AOA and AOB that occurred in response to the treatments. Since the soil was kept at constant moisture and regularly mixed, the additional disturbance introduced by the soil sampling can be considered as minimal. Fresh soils samples were used for the gross nitrification assay, whereas a portion of the sample was freeze-dried immediately after sampling and stored frozen (-20°C) until DNA extraction.

### DNA Extraction

The frozen soil samples were pre-homogenized by shaking in a MM2 ball mill (Retsch GmbH & Co. KG, Haan, Germany) at maximum speed for 3 min prior to DNA extraction. DNA was extracted from 250 mg soil using PowerSoil^TM^ DNA Isolation Kit (Mo Bio Laboratories, Inc., Carlsbad, CA, USA) according to the manufacturer’s instructions. Bead-beating was performed using the MM2 ball mill at maximum speed for 10 min. DNA was quantified spectrophotometrically using Quant-iT^TM^ PicoGreen^®^ dsDNA Assay Kit (Invitrogen, Molecular Probes Inc., Eugene, OR, USA).

### Quantitative PCR

Potential inhibitory effects on quantitative real-time PCR (qPCR) performance were initially tested on all samples by adding a known amount of the circular pGEM^®^-T plasmid (Promega Corporation, Madison, WI, USA) to sample DNA and then amplifying the plasmid with the plasmid-specific T7 and SP6 primers. The results were comparable to those obtained from controls with water and the plasmid. Thus, no inhibition of the amplification reactions was detected with the amount of DNA used in the assays described below.

For quantitative estimation of the archaeal and bacterial *amoA* genes the primers CrenamoA23f (5′-ATGGTCTGGCTWAGACG-3′) and CrenamoA616r (5′- GCCATCCATCTGTATGTCCA-3′) were used for AOA (Tourna et al., 2008) and *amoA*-1F (5′-GGGGTTTCTACTGGTGGT-3′) and *amoA*-2R (5′-CCCCTCKGSAAAGCCTTCTTC-3′) for AOB (Rotthauwe et al., 1997). Each reaction mixture (15 μl total volume) contained 7.5 μl BioRad iQ^TM^ SYBR^®^ Green Supermix (Bio-Rad Laboratories, Inc, Hercules, CA, USA), 0.5 μM of each primer, 10 μg bovine serum albumin (10 mg ml^-1^ New England Biolabs Inc.), DNA free water and 4–12 ng template DNA. Amplification of each gene was performed in duplicate runs using the CFX96 Touch^TM^ Real-Time PCR Detection System (Bio-Rad Laboratories) and PCR conditions were 95°C for 5 min, followed by 35 cycles of 15 s at 95°C, 30 s at 55°C, 30 s (for AOB), or 40 s (for AOA) at 72°C and a final step of 10 s at 78°C at which fluorescence was acquired. The reactions were finished with a melting curve starting at 65°C with an increase of 0.5°C per 2 s up to 95°C. Results were analyzed using the software Bio-Rad CFX Manager 3.1 (Bio-Rad Laboratories).

Standard curves were generated using 10-fold dilutions of linearized plasmids (pGEM-T, Promega) containing cloned bacterial and archaeal *amoA* gene fragments amplified from soil. DNA-free water (Sigma–Aldrich) was used as negative control and the quantification resulted in null or negligible values. The efficiencies of the qPCR runs were 70.3% (*R*^2^ = 0.999) for AOA and 76.5% (*R*^2^ = 0.998) for AOB. All PCR products were checked on agarose gels to verify the correct fragment size.

### Gross Nitrification

Gross nitrification rates were estimated by the ^15^*N*-pool dilution/enrichment technique. Immediately after sampling the microcosms, two sub-samples of 15 g of soil each were individually mixed with 0.5 ml ^15^NH_4_Cl-solution (containing 30 μg ^15^*N* ml^-1^) in a disposable urine container with lid (100 ml). One of the sub-samples was immediately extracted with 50 ml 1 M KCl for 2 h on an orbital shaker at 100 rpm, whereas the other was incubated at room temperature for 24 h, after which the soil was extracted with KCl. The KCl extracts were filtered (Munktell’s Filter paper 5.5 cm) and NH_4_^+^ and NO_3_^-^ isolated from the filtrate using standard IAEA diffusion procedures (International Atomic Energy Agency [IAEA], 2001). In short, an acid trap was added to the KCl extract followed by 0.2 g MgO. The containers were closed and shaken for approximately 72 h on an orbital shaker (100 rpm). The acid traps were removed and opened, and the filter disks were dried in a desiccator. The containers were left with an open lid in the dark for 1–3 days to let residues of ammonia evaporate. A new trap was added followed by 0.2 g of Devarda’s alloy and 0.2 g MgO. The containers were shaken for another 72 h and the traps were removed as above.

The dried filter disks were placed in tin cups and analyzed for ^15^*N*/^14^*N* concentrations at the stable isotope facility at the Department of Biology, Lund University. Samples were flash-combusted in a Flash 2000 elemental analyzer (Thermo Scientific Inc., Bremen Germany). The total amount of nitrogen in the filter disks was determined using the elemental analyzer’s thermal conductivity detector, and the isotopic ratios by a Delta V Plus isotope-ratio mass spectrometer connected to the elemental analyzer via the ConFlow IV interface (Thermo Scientific Inc., Bremen, Germany). The gross nitrification rate was then calculated as in Bengtson et al. (2006) using the equations in **Supplementary Table S1**. The calculation was based on three replicates since one acid trap per treatment had to be discarded.

### Statistical Analysis

To account for the fact that the same microcosms were sampled multiple times we used repeated-measures ANOVA for determining differences among treatments and sampling occasions. All statistical analyzes were performed in STATISTICA version 12 (StatSoft, Inc, Tulsa, OK, USA. 2013). The relationship between AOA:AOB ratio and gross nitrification was tested using the general regression module in STATISTICA.

## Results

### Soil Properties

The initial soil pH value of 6.5 decreased in all treatments during the experiment (*F* = 22.66, *p* < 0.001) and there were differences between the treatments (*F* = 23.30, *p* < 0.001). As expected, ammonium addition resulted in the lowest pH (5.3), whereas the other treatments reached a pH of 5.8–6.1. The concentration of ammonium was significantly higher in the soil where nitrogen (ammonium or amino acids) was added, compared to the sugar and control treatment (*F* = 147.18, *p* < 0.001, **Table 1**). Concentrations of nitrate also increased significantly when nitrogen was added as well as in the control treatment (*F* = 175.19, *p* < 0.001, **Table 1**).

**Table 1:** Concentrations of ammonium and nitrate, gross nitrification rates and the minimum and maximum contribution of ammonia-oxidizing bacteria (AOB) to the observed gross nitrification rates in different treatments and sampling occasion.

Treatment	Sampling (Day)	Concentration of ammonium [μg *N* (g dry soil)^-1^]	Concentration of nitrate [μg *N* (g dry soil)^-1^]	Gross nitrification rate [μg *N* (g dry soil)^-1^ day^-1^]	Lowest AOB contribution to nitrification (%)^a^	Highest AOB contribution to nitrification (%)^b^
Sugar	5	1.8 (0.2)	11.5 (0.3)	1.3 (0.1)	100	100
	19	1.5 (0.1)	15.9 (1.1)	8.5 (1.4)	34	60
	33	2.2 (0.4)	15.9 (0.5)	4.2 (0.8)	70	100
Amino acids	5	8.2 (0.1)	29.6 (1.4)	2.8 (0.6)	73	100
	19	27.0 (1.6)	36.8 (0.6)	4.0 (0.4)	81	100
	33	33.8 (0.4)	34.2 (1.6)	8.8 (0.6)	67	100
Ammonium	5	15.7 (0.9)	28.1 (1.0)	2.9 (0.6)	94	100
	19	30.0 (1.7)	33.2 (1.3)	11.4 (0.1)	69	100
	33	32.6 (3.5)	31.5 (1.0)	23.1 (1.2)	76	100
Control	5	2.9 (0.5)	15.5 (1.0)	3.0 (0.4)	100	100
	19	1.9 (0.3)	27.7 (3.2)	11.5 (1.2)	25	44
	33	3.7 (0.3)	34.6 (2.3)	11.9 (0.9)	25	44


*Values in brackets represent standard error (*n* = 3).*

*^a^Based on the lowest detected AOB specific nitrification rate, which was 1.5 pg *N* day^-1^ (AOB amoA copy)^-1^, found between day 5 and 19 in the amino-acid treatment.*

*^b^Based on the greatest detected AOB specific nitrification rate, which was 2.7 pg *N* day^-1^ (AOB amoA copy)^-1^, found between day 19 and 33 in the amino-acid treatment.*

### Abundance of Ammonia-oxidizing Archaea and Bacteria

**FIGURE 1:**
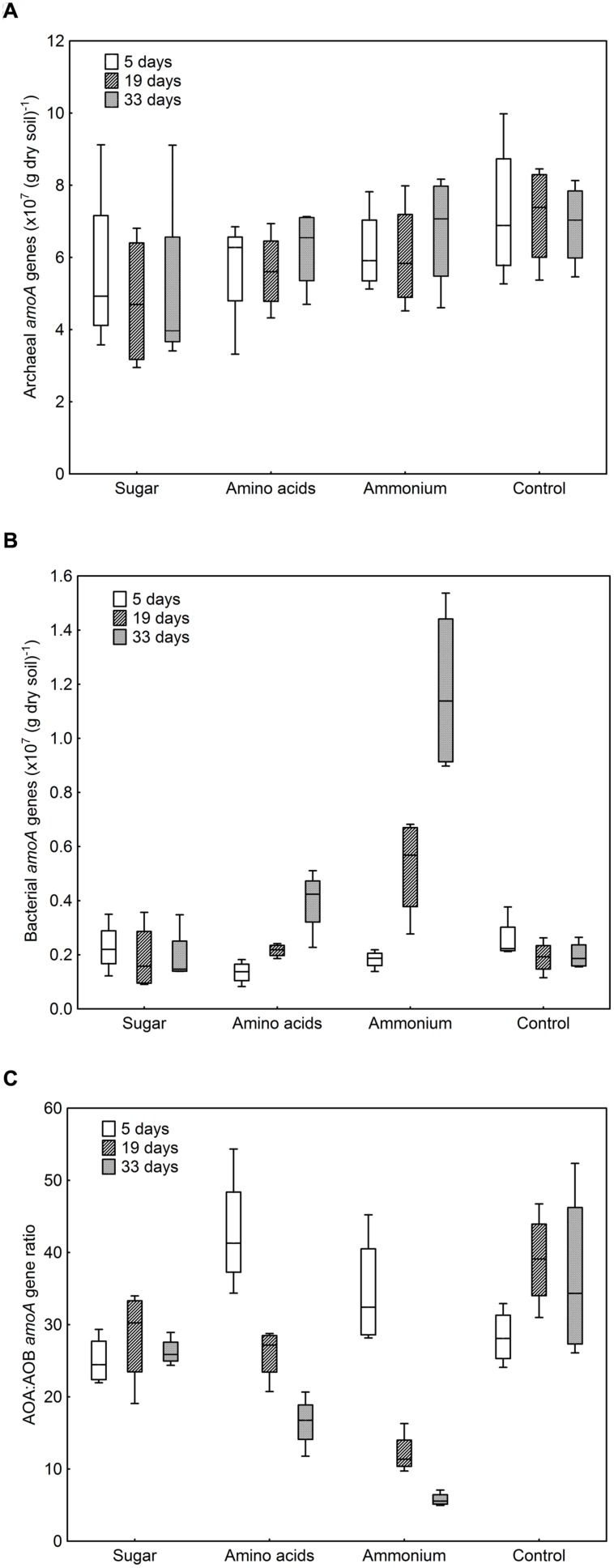
**Abundance of ammonia-oxidizing archaea (AOA) and ammonia-oxidizing bacteria (AOB) in different treatments after 5, 19, and 33 days of incubation.** **(A)** Archaeal *amoA* genes copies per g dry soil. Treatment: *F* = 2.47, *p* = 0.11; sampling time: *F* = 0.20, *p* = 0.82. **(B)** Bacterial *amoA* genes copies per g dry soil. Treatment: *F* = 74.85, *p* < 0.001; sampling time: *F* = 16.72, *p* < 0.001; interaction *F* = 11.40, *p* < 0.001. **(C)** Ratio between archaeal and bacterial *amoA* genes. Treatment: *F* = 19.07, *p* < 0.001; sampling time: *F* = 13.54, *p* < 0.001; interaction *F* = 10.86, *p* < 0.001. Boxes represent the 25 and 75% percentiles, whiskers the 5 and 95% percentile and the horizontal line in each box the median value.

The numbers of archaeal *amoA* gene copies per g dry soil only varied between 4.8 × 10^7^ and 7.2 × 10^7^, whereas the numbers of bacterial *amoA* gene copies showed a much broader range (1.3 × 10^6^–1.2 × 10^7^; **Figures 1A,B**). In the control and sugar treatment, the abundance of bacterial *amoA* genes remained constant, with a tendency to decrease with time, whereas a significant increase was observed when ammonium or amino acids were added (*F* = 16.72, *p* < 0.001, **Figure 1B**). The effect was more pronounced when ammonium was added compared to the amino-acid treatment, even though the same amount of nitrogen was added. By contrast, there was no difference in archaeal *amoA* gene copy numbers between treatments (*F* = 2.47, *p* ≥ 0.05) or over time (*F* = 0.20, *p* ≥ 0.05, **Figure 1A**).

The number of archaeal *amoA* genes exceeded that of bacterial *amoA* genes in all treatments with a ratio between AOA and AOB *amoA* genes varying between 5.8 and 42.8 (**Figure 1C**). Due to the increase in AOB when nitrogen was added, the AOA:AOB *amoA* gene ratio decreased during the cause of the experiment in these treatments. At the last sampling, the ratio was lowest in the ammonium treatment and highest in the control (**Figure 1C**).

### Contribution of AOA and AOB to Gross Nitrification

**FIGURE 2:**
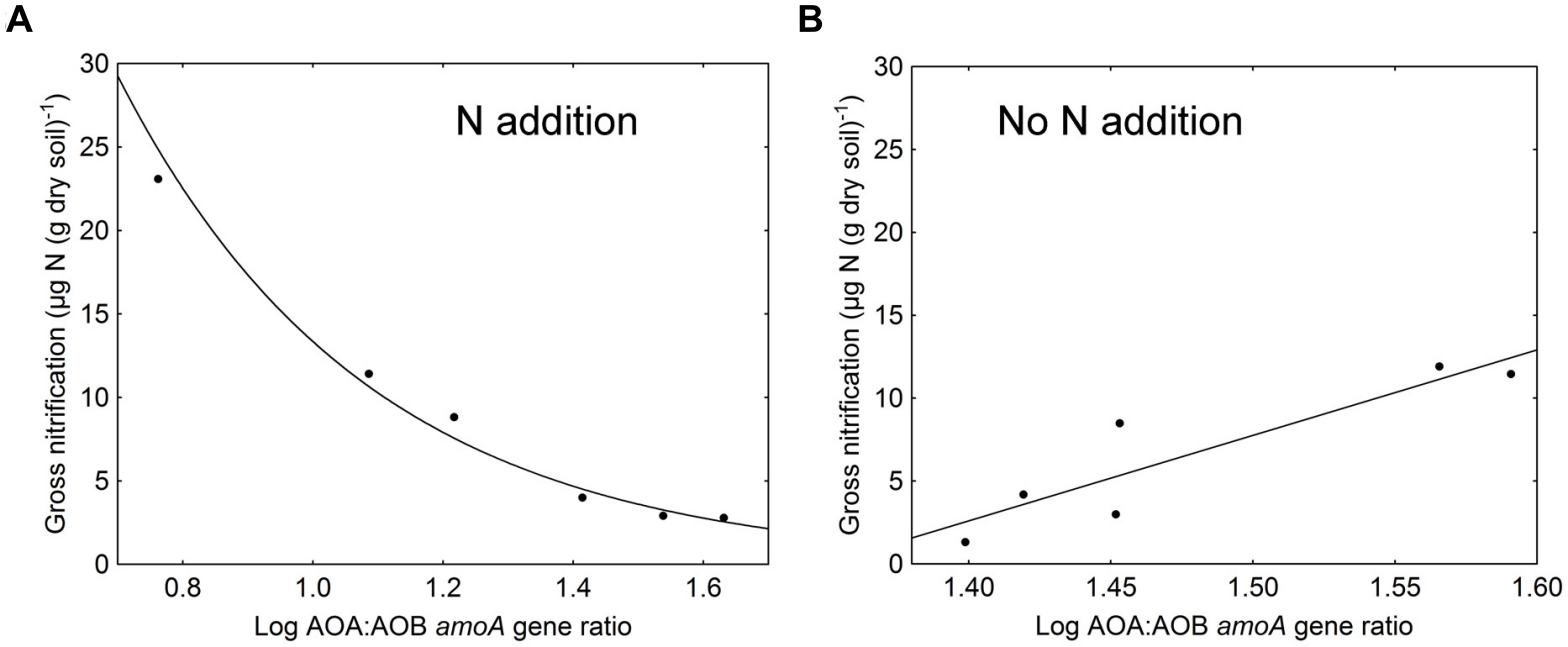
**Relationship between log-transformed AOA:AOB *amoA* gene ratios and gross nitrification rates in **(A)** treatments where nitrogen was added (amino acids and ammonium; *r* = 0.97, *p* = 0.001) and **(B)** treatments where no nitrogen was added (sugar and control; *r* = 0.91, *p* = 0.013)**.

In general, gross nitrification rates increased during the cause of the experiment (*F* = 156.72, *p* < 0.001). The largest increase and the highest nitrification rates were found in the ammonium treatment (**Table 1**). The approximately eightfold increase in gross nitrification between the first and the last sampling in the ammonium treatment corresponded to the increase in bacterial *amoA* copy numbers (approximately sixfold, **Figure 1B**). Similarly, the threefold increase in nitrification rates in the amino-acid treatment corresponded with the increase in bacterial *amoA* copy numbers of similar magnitude (from 1.4 × 10^6^ to 4.2 × 10^6^ copies per g dry soil, **Figure 1B**). Accordingly, there was a significant negative relationship between the AOA:AOB *amoA* gene ratio and the gross nitrification rate in the microcosms where nitrogen was added (*p* = 0.001; **Figure 2A**). By contrast, we found a positive relationship between the ratio of AOA:AOB *amoA* genes and the gross nitrification rate in treatments that did not receive nitrogen (*p* = 0.013; **Figure 2B**). These findings suggest that AOB dominated nitrification in the treatments where nitrogen was added, and that the relative contribution of AOA to nitrification was higher in the treatments not receiving nitrogen, in which the ammonium concentration remained low [<4 μg *N* (g dry soil)^-1^] throughout the experiment (**Table 1**).

An increase in the abundance of autotrophic AOB must by necessity be accompanied by high enough ammonia oxidation rates to support the observed increase. The amount of ammonia that needs to be oxidized is predictable and dependent on the growth yield, i.e., the amount of biomass produced (or as in this case AOB *amoA* copies) per unit of substrate (ammonia) reacted (Blackburne et al., 2007). From this data the cell-specific nitrification rate can be inferred and used to calculate the proportion of the observed nitrification that can be assigned to AOB in a certain treatment at a certain time point (Eqs 1 and 2). The minimum (Eq. 1) and maximum (Eq. 2) contribution of AOB was calculated from the lowest detected (ΔN/ΔB)_min_ and greatest detected (ΔN/ΔB)_max_ AOB specific nitrification [pg NH_4_^+^-*N* day^-1^ (AOB *amoA* copy)^-1^]. This was calculated from the lowest and greatest change in nitrification rate (ΔN) and abundance of bacterial *amoA* gene copies (ΔB) between two sampling times. The lowest AOB specific nitrification [1.5 pg NH_4_^+^-*N* day^-1^ (AOB *amoA* copy)^-1^] was detected between day 5 and 19 in the amino-acid treatment and the greatest (2.7 pg NH_4_^+^-*N* day^-1^ (AOB *amoA* copy)^-1^] between day 19 and 33 in the amino-acid treatment.

$${\left(\Delta N/\Delta B\right)}_{\min}\times B]/N\times 100=\min imum\,\,contribution\,\,of\,\,AOB\,\,to\,\,nitrification\left(\%\right)$$

$${\left(\Delta N/\Delta B\right)}_{\max}\times B]/N\times 100=\max imum\,\,contribution\,\,of\,\,AOB\,\,to\,\,nitrification\left(\%\right)$$

where *N* is the gross nitrification rate [μg *N* (g dry soil)^-1^], B the number of bacterial *amoA* genes [copies (g dry soil)^-1^], (ΔN/ΔB)_min_ = 1.5 pg NH_4_^+^-*N* day^-1^ (AOB *amoA* copy)^-1^, and (ΔN/ΔB)_max_ = 2.7 pg NH_4_^+^-*N* day^-1^ (AOB *amoA* copy)^-1^.

Based on these calculations, we conclude that if all the AOB present in the soil oxidize ammonia at maximum capacity they can account for all nitrification in the *N*-amended soils and in the sugar amended soil at day 5 and 33, as well as at day 5 in the control (**Table 1**). However, they cannot contribute more than 44% of the overall nitrification in the control treatment at days 19 and 33 (**Table 1**). This suggests that AOA contribute to at least 56% of the nitrification observed in the control soil.

## Discussion

Repeated additions of amino acids or ammonium resulted in increasing ammonium concentrations, bacterial *amoA* gene numbers and gross nitrification rates. The increase in nitrification during the cause of the experiment was much higher in treatments that received ammonium compared to the treatment that received amino acids, even if the same amount of nitrogen was added in both treatments. This might be explained by a poor competitive ability of nitrifiers for nitrogen in relation to heterotrophs (Verhagen and Laanbroek, 1991; Verhagen et al., 1995), with the latter being favored by the addition of amino acids that also serve as an energy source. Nevertheless, the increase in gross nitrification was of similar magnitude as the increase in bacterial *amoA* genes in both nitrogen treatments. Since there was no concurrent increase in the abundance of archaeal *amoA* genes, the increased nitrification mainly seems to be a result of the increased abundance of nitrogen responsive AOB. This is supported by other studies that show that at least a part of the AOB community is favored by high ammonium concentrations (Di et al., 2009, 2010; Jia and Conrad, 2009; Verhamme et al., 2011). In contrast to the nitrogen-amended soils, the ammonium concentration remained low throughout the experiment in the sugar treatment and control. The decreased nitrification rate at the last sampling in the sugar-amended soil is likely an effect of the nitrogen-poor environment created by the repeated addition of carbon, which would result in competition for inorganic nitrogen made available through mineralization between ammonia oxidizers and heterotrophs (Verhagen and Laanbroek, 1991; Verhagen et al., 1995). This reasoning is supported by the low nitrate concentrations detected in this treatment. It has been suggested that nitrogen-poor conditions favor AOA rather than AOB (Offre et al., 2009; Sauder et al., 2012). Accordingly, we observed a positive relationship between the AOA:AOB *amoA* gene ratio and gross nitrification in treatments without nitrogen additions. Altogether, these findings support previous conclusions that AOA and AOB generally inhabit different niches in soil separated by ammonium concentration and availability (Verhamme et al., 2011; Wessén et al., 2011), and further builds on the findings by demonstrating that the influence of ammonia concentrations on AOA and AOB is not only limited to affecting their abundance and composition, but also their activity.

Even though AOB were the dominant ammonia oxidizers in the ammonium treatment and bacterial *amoA* gene copies increased nearly 10-fold in this treatment, they were still recorded at lower numbers than the archaeal *amoA* genes. In fact, we detected between 6 and 43 times more *amoA* genes of archaeal origin than bacterial across all treatments. Assuming that each AOB has 2.5 copies of the *amoA* gene (Norton et al., 2002) and AOA only one copy (Hatzenpichler, 2012 and references therein), this corresponds to an AOA:AOB cell ratio of 15–108. Our estimations of the abundance of AOA and AOB, combined with measurements of gross rather than net nitrification rates, enabled us to calculate the minimum contribution of AOA to ammonia oxidation. By estimating the maximum proportion of the gross nitrification that can be attributed to AOB, we found that AOB could not account for all of the nitrification activity in the control treatment (days 19 and 33) and in the sugar-amended soil at day 19. In fact, a minimum of 40–56% of the activity could be attributed to AOA, providing evidence that AOA dominate not only in numbers, but also contribute substantially to the nitrification process under these conditions. These findings suggest that AOA can strongly contribute to ammonia oxidation not only in acidic soils (He et al., 2012), but also in soil with a near neutral pH. However, since the abundance of AOA was higher than that of AOB, the cell-specific nitrification rate for AOA on average appears much lower than that of AOB (assuming that all AOA detected in the soil are actively oxidizing ammonia). This is supported by previous estimations of 0.53 fmol NH_3_ cell^-1^ h^-1^for the AOA *N. maritimus* and 4–23 fmol NH_3_ cell^-1^ h^-1^ for AOB in pure cultures (Belser, 1979; Martens-Habbena et al., 2009). If we assume that AOB alone were responsible for the increase in gross nitrification in the nitrogen-amended microcosms, the cell-specific ammonia oxidation rate of AOB was 11–20 fmol NH_3_ (AOB cell)^-1^ h^-1^, which corresponds well to the rates from pure cultures. In the control treatment where AOA had a minimum contribution of 56% to the observed nitrification, the cell specific rate was calculated to a minimum of 0.27–0.29 fmol NH_3_ (AOA cell)^-1^ h^-1^. Jia and Conrad (2009) estimated the cell-specific rates for AOA and AOB in an agricultural soil to 0.005–0.738 fmol NH_3_ cell^-1^ h^-1^ and 0.25–13.5 fmol NH_3_ cell^-1^h^-1^, respectively (based on nitrate production). Thus, both pure-culture data and environmental estimates show that the cell-specific activity for AOB is 10 times higher or more than for AOA, probably due to smaller cell size for AOA (Prosser and Nicol, 2012). However, all archaea carrying the *amoA* gene might not only gain energy from oxidation of ammonia, but rather persist through a mixotrophic or even heterotrophic lifestyle (Hallam et al., 2006; Mußmann et al., 2011).

It has been suggested that the origin of the ammonium could be an important factor influencing which group of ammonia-oxidizing microorganisms that will be responsible for nitrification. Both Levičnik-Höfferle et al. (2012) and Chan et al. (2013) showed that AOA responded with an increase in *amoA* gene copy numbers to organic nitrogen, but not to inorganic nitrogen fertilization. Our results do not fully support these ideas since bacterial, but not archaeal *amoA* genes increased in abundance irrespective of if nitrogen was added in organic or mineral form. On the other hand, when sugar or water was added the substrate for the ammonia oxidizers was only supplied by mineralization of soil organic matter, which has been suggested to favor AOA (Stopnišek et al., 2010; Wertz et al., 2011; Habteselassie et al., 2013). Consequently, in these treatments we found a positive relationship between the ratio of AOA:AOB *amoA* genes and gross nitrification. The observation that the relatively low numbers of AOB could not alone account for the measured ammonia oxidation rates supports that AOA had to contribute to the process in these treatments. This can also explain the increase in the AOA:AOB *amoA* gene ratio with time in the control treatment.

The ammonia-oxidizing community in this grassland soil was dominated by AOA. Archaeal *amoA* genes outnumbered the bacterial counterpart by up to 40-fold. Nitrogen additions resulted in an increase in bacterial *amoA* genes, suggesting a stimulation of AOB but not AOA, and a positive relationship between the AOB *amoA* gene abundance and gross nitrification rates were observed in these treatments. However, in non-nitrogen supplemented soils, the abundance of AOB was relatively low, and they could not account for the observed nitrification rates. This is supported by the positive relationship between AOA:AOB ratios and gross nitrification rates under these conditions. Altogether, our results demonstrate that input of easily available nitrogen favored AOB-driven nitrification even if AOA dominated in numbers in this pH neutral grassland soil, but that AOA were important for nitrification under nitrogen-poor conditions.

## Author Contributions

AS designed the experiment with input from PB and SH. AS performed the experiment and subsequent qPCR analyses, statistical analyses, and interpretation of the results (with support from PB and SH). Quantification of AOA and AOB by qPCR was done by AS with support from SH. PB performed the isotopic analyses. AS wrote the manuscript with inputs from PB and SH.

## Conflict of Interest Statement

The authors declare that the research was conducted in the absence of any commercial or financial relationships that could be construed as a potential conflict of interest.
