# Supplementary material for: Archaeal Ammonia Oxidizers Dominate in Numbers, but Bacteria Drive Gross Nitrification in N-amended Grassland Soil
Source: Front Microbiol. 2015 Nov 30;6:1350. doi: 10.3389/fmicb.2015.01350 (PMC4663241; doi:10.3389/fmicb.2015.01350)
Supplement: Supplementary file 1 [file Table_1.PDF]

Supplementary Table 1. The equations used to calculate the gross N mineralization, immobilization, and nitrification rates, and the definition of the symbols in the equations.

Equation		Symbol	Meaning	Unit
		c	Gross NH_4^+ consumption	mg N kg $^{-1}$ day $^{-1}$
$p = \dfrac{\ln \dfrac{f_t - k}{f_0 - k}}{\ln \dfrac{W_t}{W_0}} \times \dfrac{W_0 - W_t}{t}$	(1)	f	^{15}N abundance of NH_4^+	atom %
		g	^{15}N abundance of NO_3^-	atom %
		$\overline{g}$	Average ^{15}N abundance of NO_3^-	atom %
$c = \left[1 + \dfrac{\ln \dfrac{f_t - k}{f_o - k}}{\ln \dfrac{W_t}{W_0}} \right] \times \dfrac{W_0 - W_t}{t}$	(2)	k	Natural ^{15}N abundance	atom %
		p	Gross NH_4^+ production	mg N kg $^{-1}$ day $^{-1}$
		r	Gross NO_3^- production	mg N kg $^{-1}$ day $^{-1}$
$r = \dfrac{(g_t - \overline{g})Z_t - (g_0 - \overline{g})Z_0}{(k - \overline{g})t + \dfrac{(f_0 - k)W_0}{c} - \dfrac{(f_t - k)W_t}{c}}$	(4)	t	Time	day
		W	Concentration of ^{14}N plus ^{15}N in NH_4^+	mg N kg $^{-1}$
		Z	Concentration of ^{14}N plus ^{15}N in NO_3^-	mg N kg $^{-1}$
